# A comparative study of anxiety and depression among healthcare workers and non-healthcare workers in Johor, Malaysia during the Covid-19 era

**DOI:** 10.1097/MD.0000000000037415

**Published:** 2024-03-22

**Authors:** Jing Wen Wong, Jun Hui Tan, Ruth Elizabeth Abraham, Shareen Nisha Jauhar Ali, Si Yin Kok, Henry Chor Lip Tan, Jih Huei Tan, Han Ni

**Affiliations:** a Newcastle University Medicine Malaysia, Iskandar Puteri, Malaysia; b Department of General Surgery, KPJ Bandar Dato Onn Specialist Hospital, Johor Bahru, Malaysia; c Department of General Surgery, Hospital Sultanah Aminah, Johor Bahru, Malaysia.

**Keywords:** anxiety, Covid-19, depression, healthcare workers, non-healthcare workers

## Abstract

The outbreak of Coronavirus disease 2019 (Covid-19) has a significant impact on the mental health of the global population. Updates are needed regarding the mental health status among the local population since limited studies were done so far. This research compared the prevalence of anxiety and depression symptoms among HCWs and non-HCWs. We also evaluated the factors associated with anxiety and depression symptoms among these 2 groups. This was a cross-sectional study conducted between September to December 2022. Online questionnaire was distributed to HCWs from 2 tertiary government hospitals. Non-HCWs from various occupational fields were recruited randomly. Generalised Anxiety Disorder 7 (GAD-7) and Patient Health Questionnaire 9 (PHQ-9) were used to screen for anxiety and depression symptoms respectively. Data were analyzed using IBM SPSS version 28.0. 200 questionnaires were distributed to HCWs and non-HCWs respectively. The response rate was 74.5% from HCWs and 82.5% from non-HCWs (*P* = .07). A total of 236 individuals (105 HCWs and 131 non-HCWs) were included in the study. Majority were female, married, highly educated and worked more than 8 hours per day. There was no significant difference for the prevalence of anxiety (37.2% vs 44.3%, *P* = .34) and depression symptoms (37.3% vs 35.1%, *P* = .75) between HCWs and non-HCWs. Among HCWs, poor workplace support (*P* = .009) and low income (*P* = .04) were associated with anxiety symptoms. Younger age (*P* = .02), single status (*P* = .01) and poor workplace support (*P* = .006) were associated with depression symptoms. More non-HCWs with a higher educational level were having anxiety and depression symptoms. Single status (*P* = .03), working away from home (*P* = .02), poor family support (*P* = .03) and quarantine as Covid-19 close contact (*P* = .04) were also associated with depression symptoms among non-HCWs. There is no significant difference between HCWs and non-HCWs experiencing possible anxiety or depressive symptoms in this study. However, attention should be paid to address associated factors identified among each group to promote good mental health.

## 1. Introduction

The first case of coronavirus disease 2019 (Covid-19) was reported in Wuhan City, Hubei Province of China when a case of atypical pneumonia of unknown origin was identified on December 31, 2019. In the span of just 4 days, a further 44 cases were reported by the national authorities of China.^[1]^ Since then, the virus has spread rapidly to other countries across the world. The widespread transmission of this contagious virus led the World Health Organisation (WHO) to initially declare Covid-19 as a Public Health Emergency of International Concern (PHEIC) on January 30, 2020. On March 11, 2020, it was officially declared a pandemic.^[2–4]^ As of January 14, 2024, a total of 701,686,303 cases of Covid-19 and 6,968,402 deaths have been reported worldwide.^[5]^ The first Covid-19 case involving a Malaysian was reported on February 04, 2020.^[6]^

Studies have shown that the pandemic has had a negative impact on the publics’ mental health, leading to an upsurge in the prevalence of anxiety and depression across the world.^[7–10]^ The pandemic has also put major strains on the Malaysian healthcare system. The lack of proper evidence-based diagnostic and therapeutic measures during the initial period took a toll on healthcare professionals. They were forced to adopt a trial-and-error approach in treating patients, as the disease quickly infected millions and claimed the lives of thousands of people daily. During the initial stages, healthcare professionals had to work round the clock, delivering the best treatment that they could offer without knowing the exact pathology of the disease nor the optimal treatment. There were also uncertainties surrounding the duration of infectivity and the period of isolation required.^[11]^ Furthermore, in critical Covid-19 cases, patients with severe hypercapnia secondary to type 2 respiratory failure required ventilators. However, the limited ICU beds and ventilators failed to meet this surge in demand.^[6]^

Owing to the increasing number of daily cases, the Malaysian government decided to implement Movement Control Order (MCO) on March 18, 2020.^[6]^ Social isolation had become the new norm and the public was not prepared for the repercussions of such a decision. These restrictions also had massive adverse effects on both economic and educational sectors. During the MCO period, it was noted that Malaysia was accumulating a loss of RM 2.4 billion a day.^[11]^ As a result, Malaysians faced high levels of anxiety and significant psychological distress due to their inability to cope with this abrupt change.^[12]^

Considering these reasons, it is logical and reasonable to deduce that healthcare workers (HCWs) probably endure greater psychological distress compared to non-healthcare workers (non-HCWs). This postulation is supported by multiple studies across the globe.^[13–17]^ However, some studies shown that the prevalence of mental health problems was lower among HCWs.^[18–20]^ This was explained by the fact that HCWs, especially frontliners, were better prepared mentally and worked well under high pressure situations. In the context of association factors, females were found to be more vulnerable to psychological problems during the Covid-19 era.^[15,21]^ Moreover, younger people are also at higher risk of developing mental health problems.^[9,21]^ The same study also concluded that preexisting chronic health conditions, and a reduction in income during social isolation period contributed to the psychological stress faced by the general population.^[9]^

The implications of Covid-19 are long-lasting and adversely affect the general population up till this day. Holmes et al published a paper in The Lancet, urging more studies looking into the psychological impacts caused by Covid-19 to be conducted.^[22]^ Limited studies have been carried out in Johor, one of the bigger states in Malaysia with a larger population. Therefore, this study aims to compare the prevalence of anxiety and depression symptoms between HCWs and non-HCWs in Johor during the Covid-19 era.

Primary prevention is vital to curb the surge in mental health conditions.^[23,24]^ Mental health promotion interventions have been proven to improve mental health status, reducing the incidence of mental health disorders and strengthening an individual’s ability to adapt adversity.^[23]^ Identification of associated factors will enable selective prevention programmes that tackle modifiable risk factors and enhance protective factors in the development of mental health disorders.^[24]^ By identifying the associated factors in HCWs and non-HCWs, we hope to promote awareness and encourage mental health promotion, surveillance, and interventions targeted at individual, community, and national levels.

## 2. Methods

This research was a cross-sectional survey carried out between September to December 2022 using a self-administered questionnaire. Validated GAD-7 and Patient Health Questionnaire 9 (PHQ-9) scales were integrated in the questionnaire to screen for anxiety and depression symptoms. The survey was piloted on 10 subjects to test its validity, and these data were not included in the final analysis. The study participants were the working adults in Johor. HCWs were selected using a purposive sampling method to retrieve an accurate and representative target population. Purposive sampling is a non-probability sampling technique.^[25]^ Defined criteria were applied for the conscious selection of study subjects based on the researcher’s expertise and knowledge of the subjects.^[25]^ Hospital Sultanah Aminah and Hospital Sultan Ismail are the main referral points for various secondary and district healthcare centers across Johor. Hence, HCWs from these 2 centers were selected as the target population in this study. As for non-HCWs, earning individuals from any occupational field in Johor were recruited randomly. The following exclusion criteria were applied: individuals who are not in Johor, not currently working or those diagnosed with depression or anxiety before December 31, 2019, which was before the start of Covid-19 pandemic.

The survey was conducted via Google form as it is a convenient and environmentally friendly tool to collect data from the participants. The submission was anonymous, and was set to gain the respondents’ informed consent before filtering the respondents through the exclusion criteria. All questions were phrased in a simple English language to ensure the participants can understand and provide valid responses. The survey was disseminated via social media platforms such as Facebook and WhatsApp. A brief introductory text was provided before the individual decided whether to click on the Google form link. In the form, declarations about anonymity and confidentiality were re-emphasized. The participants gave their informed consent by clicking “Yes” and providing the date of consent. If they clicked “No,” they will be led to the submission page to end the survey. Our team personally approached a minority of participants with technical issues to assist them in completing the questionnaire. There was no incentive or compensation for the participants involved.

A formula was used to calculate the estimated sample size for the research.^[26]^ The exact number of working adults in Johor is unknown, hence the number of the whole Johor population, 3,790,000 was used.^[27]^ To the time of research conduction, there was no similar study on the same target population. According to the National Health and Morbidity Survey 2015, 29% of Malaysian adults have mental disorders.^[28]^ A 2021 study on 326 Malaysians reported a prevalence of 23.9% for anxiety symptoms and 41.7% for depression symptoms.^[29]^ In view of these, the anticipated frequency was put as 30%. The confidence limit was set as 5%, and the design effect was set as 1.0. A confidence level of 95% was chosen. Hence, the estimated sample size is 323.

Sociodemographic data (age, gender, race, marital status, education level, income, familial support, number of dependents) were collected from the participants as self-reported measures. These factors have widely recognized associations with increased susceptibility to depression and anxiety, particularly in the context of Covid-19 pandemic.^[7–10,13,14,19,30–32]^ A Scotland cross-sectional survey revealed significant associations between demographic factors and the onset of anxiety and depression amidst the pandemic.^[30]^ Numerous studies have consistently highlighted female gender as the strongest predictor for the development of anxiety and depression.^[7,17,21]^ A probability sample survey of the UK population elicited that young women and individuals living with young dependents were more at risk of anxiety and depression during the Covid-19 era.^[31]^

Both HCWs and non-HCWs were asked to choose their category of profession according to the International Standard Classification of Occupation (ISCO-08).^[33]^ They were also enquired about their working condition (working hours, location, workplace support) and health status (height, weight, BMI, preexisting physical health condition, preexisting mental health conditions except anxiety and depression diagnosed before December 31, 2019). Working conditions are pivotal in understanding mental health outcomes, given its pervasive impact on adult life.^[34,35]^ A systematic review identified poor health and lifestyle choices, unsupportive workplace relationships, as well as excessive job demands as primary risk factors for anxiety and depression.^[35]^

A recent Pakistan study showed that having Covid-19 symptoms significantly impacted the anxiety level of both HCWs and non-HCWs.^[36]^ As for Covid-19 related events, its significance in relation to the development of mental health conditions remains inadequately explored in existing literature. Hence, we decided to integrate a series of Covid-19 related events (history of being quarantined as close contact, contracting Covid-19, hospitalization due to Covid-19 and family history of Covid-19) in the survey.

Participants’ mental health status was assessed using the validated GAD-7 and PHQ-9 scales. They rated each item in the scales as 0 (not at all), 1 (several days), 2 (more than half the days) or 3 (nearly every day) based on their past 2 weeks’ experience. GAD-7 is a 7-item anxiety scale to screen and assess the severity of anxiety symptoms. It has good reliability in both self-report and interviewer-administered versions, with a sensitivity of 89% and specificity of 82%.^[37]^ The score can range from 0 to 21. GAD-7 scores of 5 or more, 10 or more and 15 or more suggest mild, moderate, and severe anxiety respectively.^[37]^ PHQ-9 is a 9-item depression scale that can be reliably self-administered to screen for depression.^[38]^ It has a sensitivity of 88% and specificity of 88% for major depression.^[38]^ The score can range from 0 to 27. The participants’ symptoms were classified into mild (score of 5 or more), moderate (10 or more), moderately severe (15 or more), or severe (20 or more).^[38]^ Only the English version of both GAD-7 and PHQ-9 were used.

In the questionnaire, information was provided about the scoring method of GAD-7 and PHQ-9. Participants were able to sum up their scores and understand more about their current mental health status. Aside from collecting data from the participants, we also hoped to encourage those in need to seek help for detailed assessment. To maintain confidentiality, a paragraph was included on the submission page that urged participants who scored moderate and above to seek proper medical attention. Helplines for counseling services were also attached for reference.

The study was conducted with ethical principles outlined in the Declaration of Helsinki and Malaysian Good Clinical Practice Guideline. Ethical approval was obtained from the institutional research board (IRB) of Newcastle University Medicine Malaysia. The Malaysian National Medical Research Register ID for this research is 22-01794-SQL. Online informed consent was sought from each participant prior to data collection. Participation was fully voluntary.

Data analysis was performed using IBM SPSS Version 28 (Armonk, New York, USA). Descriptive analyses were reported as frequencies and percentages. Chi-square test was used to assess for significant difference in prevalence between HCWs and non-HCWs.^[39]^ Chi-square test and odds ratio was used to determine the contributing factors in HCWs and non-HCWs.^[39]^ The confidence interval was 95% and a *P* value of < .05 was considered significant.

## 3. Results

A total of 236 individuals (105 HCWs and 131 non-HCWs) were included in the study. Majority were female, married, highly educated and worked more than 8 hours per day. There was no significant difference for the prevalence of anxiety (37.2% vs 44.3%, *P* = .34) and depression symptoms (37.3% vs 35.1%, *P* = .75) between HCWs and non-HCWs. Among HCWs, poor workplace support and low income were associated with anxiety symptoms. Younger age (*P* = .02), single status (*P* = .01) and poor workplace support (*P* = .006) were associated with depression symptoms. More non-HCWs with a higher educational level were having anxiety and depression symptoms. Single status (*P* = .03), working away from home (*P* = .02), poor family support (*P* = .03) and quarantine as Covid-19 close contact (*P* = .04) were also associated with depression symptoms among non-HCWs.

Two hundred questionnaires were distributed to HCWs and non-HCWs respectively, with a response rate of 74.5% from HCWs and 82.5% from non-HCWs (*P* = .07). A total of 314 individuals consented to participate in the study. 78 participants were excluded based on the exclusion criteria mentioned. Hence, there were 236 valid responses where all the questions were answered completely. 105 were HCWs and 131 were non-HCWs. The majority were female (72.4% HCWs and 64.9% non-HCWs, *P* = .26). There were more HCWs in the younger age group compared to non-HCWs (64.8% vs 42.7%, *P* = .001). Malay and Chinese made up the largest group of HCWs (58.1%) and non-HCWs (53.4%) respectively. Most participants were highly educated (88.6% HCWs and 71.0% non-HCWs, *P* = .001) and have low or middle income (*P* = .04). Participants’ income was divided into 3 categories according to the 2022 Household Income Survey Report Johor, namely low income (B40, monthly income < RM 5740 or 1214.2 USD), middle income (M40, monthly income ≤ RM 11919 or 2521.2 USD) and high income (T20, monthly income ≥ RM 11920 or 2521.4 USD).^[40]^

**Figure 1.**
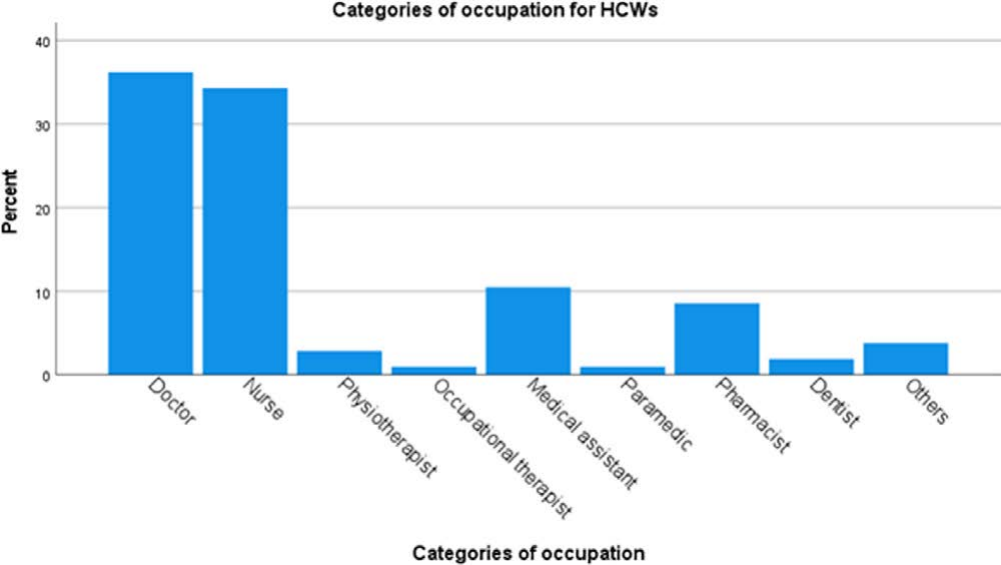
Occupational categories of healthcare workers (HCWs). Doctors and nurses made up the largest proportion of HCWs recruited (36.2% and 34.3% respectively).

**Figure 2.**
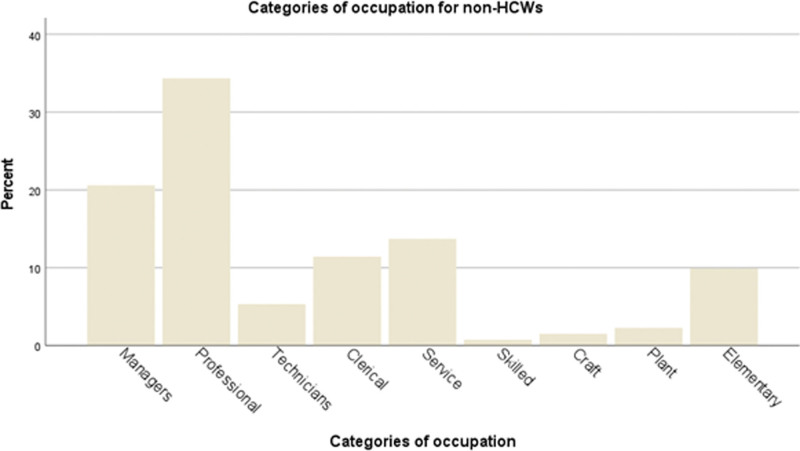
Occupational categories of non-healthcare workers (non-HCWs). The 2 most common occupational subgroups were professionals (34.3%) and managers (20.6%).

The participants’ occupation was shown in Figure 1 (HCWs) and Figure 2 (non-HCWs) according to the International Standard Classification of Occupation (ISCO-08).^[33]^ Doctors and nurses made up the largest proportion of HCWs recruited (36.2% and 34.3%) (Fig. 1). For non-HCWs, the 2 most common occupational subgroups were professionals (34.3%) and managers (20.6%) (Fig. 2). Full characteristics of the participants are shown in Table 1. Most respondents worked more than 8 hours per day (87.6% HCWs and 70.2% non-HCWs, *P* = .001) and worked from office (93.3% HCWs and 72.5% non-HCWs, *P* < .001). They generally received good workplace support. Only a minority of them lost their jobs during Covid-19 (4.8% HCWs and 7.6% non-HCWs, *P* = .43).

**Table 1:** Characteristics of participants.

	HCWs* (n = 105) n (%)	Non-HCWs† (n = 131) n (%)	*P* value
A. Sociodemographic			
Gender			
Male	29 (27.6)	46 (35.1)	.26
Female	76 (72.4)	85 (64.9)	
Age			
≤40	68 (64.8)	56 (42.7)	.001
>40	37 (35.2)	75 (57.3)	
Race			
Malay	61 (58.1)	44 (33.6)	<.001
Chinese	30 (28.6)	70 (53.4)	
Indian	14 (13.3)	16 (12.2)	
Others	0 (0)	1 (1.8)	
Marital status			
Single/divorced/widowed	33 (31.4)	44 (33.6)	.78
Married	72 (68.6)	87 (66.4)	
Education			
Lower educational level	12 (11.4)	38 (29.0)	.001
Higher educational level	93 (88.6)	93 (71.0)	
Income			
Low (B40‡)	55 (52.4)	60 (45.8)	.04
Middle (M40§)	32 (30.5)	59 (45.0)	
High (T20‖)	18 (17.1)	12 (9.2)	
Family support			
Good	97 (92.4)	116 (88.5)	.38
Poor	8 (7.6)	15 (65.2)	
Number of dependents			
<3	21 (20.0)	20 (15.3)	.39
≥3	84 (80.0)	111 (84.7)	
B. Working condition			
Working hours			
≤8 hours per day	13 (12.4)	39 (29.8)	.001
>8 hours per day	92 (87.6)	92 (70.2)	
Working location			
Work from home	1 (1.0)	8 (6.1)	<.001
Work from office	98 (93.3)	95 (72.5)	
Hybrid	6 (5.7)	28 (21.4)	
Working away from home			
Yes	44 (41.9)	33 (25.2)	.008
No	61 (58.1)	98 (74.8)	
Workplace support			
Good	78 (74.3)	100 (76.3)	.76
Poor	27 (25.7)	31 (23.7)	
Loss of job during Covid-19			
Yes	5 (4.8)	10 (7.6)	.43
No	100 (95.2)	121 (92.4)	
C. Health status			
BMI			
Non-obese	83 (79.0)	95 (72.5)	.29
Obese	22 (21.0)	36 (27.5)	
Physical health condition			
Yes	28 (26.7)	41 (31.3)	.47
No	77 (73.3)	90 (68.7)	
Mental health condition¶			
Yes	2 (1.9)	3 (2.3)	1.0
No	103 (98.1)	128 (97.7)	
D. Covid-19 related events			
Quarantined as close contact			
Yes	73 (69.5)	84 (64.1)	.41
No	32 (30.5)	47 (35.9)	
Contracted Covid-19			
Yes	63 (60.0)	74 (56.5)	.60
No	42 (40.0)	57 (43.5)	
Management for Covid-19			
Not applicable	41 (39.0)	57 (43.5)	.15
Quarantined	61 (58.1)	74 (56.5)	
Admitted	3 (2.9)	0 (0)	
Family contracted Covid-19			
Yes	76 (72.4)	98 (74.8)	.77
No	29 (27.6)	33 (25.2)	
Family management for Covid-19			
Not applicable	29 (27.6)	33 (25.2)	.50
Quarantined	70 (66.7)	94 (71.8)	
Admitted	6 (5.7)	4 (3.1)	
Loss of family from Covid-19			
Yes	6 (5.7)	6 (4.6)	.77
No	99 (94.3)	125 (95.4)	

Data collected were divided into sociodemographic, working condition, health status and Coronavirus disease 2019 related events.

* Healthcare workers.

† Non-healthcare workers.

‡ Monthly income < RM 5740 or 1214.2 USD.

§ Monthly income ≤ RM 11919 or 2521.2 USD.

‖ Monthly income ≥ RM11920 or 2521.4 USD.

¶ A medical diagnosis of any mental health condition (except anxiety and depression) before December 31, 2019.

Regarding health status, most participants did not have preexisting physical or mental health conditions. Over half of the HCWs and non-HCWs were once quarantined as close contact (69.5% vs 64.1%, *P* = .41) and contracted Covid-19 before (60.0% vs 56.5%, *P* = .60), with only a minority requiring hospital admission for further care. Similar context was identified among their family members (Table 1).

As shown in Table 2, 37.2% (n = 39) of HCWs and 44.3% (n = 58) of non-HCWs were having anxiety symptoms, with a larger proportion having mild symptoms (22.9% vs 31.3%) and the least (3.8% vs 4.6%) having severe anxiety symptoms. For depression symptoms, the prevalence was 37.3% (n = 39) among HCWs and 35.1% (n = 46) among non-HCWs. The majority of HCWs were having mild symptoms. There was no significant difference for the prevalence between both groups, with a *P* value of .34 for possible anxiety and .75 for possible depression.

**Table 2:** Title: Prevalence of anxiety and depression symptoms.

	HCWs* (n = 105) n (%)	Non-HCWs† (n = 131) n (%)	*P* value
Possible anxiety	**39 (37.2**)	**58 (44.3**)	**.34**
Mild	24 (22.9)	41 (31.3)	
Moderate	11 (10.5)	11 (8.4)	
Severe	4 (3.8)	6 (4.6)	
Possible depression	**39 (37.3**)	**46 (35.1**)	**.75**
Mild	26 (24.8)	23 (9.7)	
Moderate	7 (6.7)	13 (5.5)	
Moderately severe	3 (2.9)	8 (3.4)	
Severe	3 (2.9)	2 (0.8)	

The overall prevalence of anxiety and depression symptoms was shown here. The prevalence of each severity was also included. Comparison between healthcare workers (HCWs) and non-healthcare workers (non-HCWs) were made.

*P* value < .05: statistical significance.

* Healthcare workers.

† Non-healthcare workers.

Income and workplace support were found to be the factors associated with anxiety symptoms among HCWs (Table 3). Individuals with low income (B40) were more likely to have anxiety symptoms (47.3% vs 28.0%, *P* = .04, OR 2.31, 95% CI 1.02–5.20) than the other 2 income groups. Poor workplace support was also associated with significantly higher prevalence of anxiety symptoms compared to good support (59.3% vs 30.8%, *P* = .01, OR 3.27, 95% CI 1.32–8.10). Although the majority of HCWs and non-HCWs received higher level of education (tertiary education and above), significant difference in the prevalence of anxiety symptoms has only been identified among non-HCWs. 50.5% of highly educated individuals were having anxiety symptoms, with only 28.9% prevalence identified in the counterpart (*P* = .02, OR 2.51, 95% CI 1.12–5.64).

**Table 3:** Associated factors for anxiety symptoms.

	HCWs* (n = 105)			Non-HCWs† (n = 131)		
	Prevalence of possible anxiety (%)	OR (95% CI)	*P* value	Prevalence of possible anxiety (%)	OR (95% CI)	*P* value
A. Sociodemographic						
Gender						
Male	44.8	1.47 (0.62–3.52)	.38	41.3	0.83 (0.40–1.71)	.62
Female	35.5	0.68 (0.28–1.62)		45.9	1.20 (0.58–2.49)	
Age						
≤40	44.1	2.13 (0.89–5.08)	.096	46.4	1.16 (0.58–2.34)	.72
>40	27.0	0.47 (0.20–1.12)		42.7	0.86 (0.43–1.72)	
Marital status						
Single‡	51.5	2.26 (0.98–5.26)	.06	54.5	1.87 (0.90–3.90)	.09
Married	31.9	0.44 (0.19–1.03)		39.1	0.53 (0.26–1.11)	
Education						
Lower level	16.7	0.29 (0.06–1.40)	.10	28.9	0.40 (0.18–0.90)	**.02**
Higher level	40.9	3.45 (0.72–16.66)		50.5	2.51 (1.12–5.64)	
Income						
Low (B40§)	47.3	2.31 (1.02–5.20)	**.04**	50.0	1.54 (0.77–3.08)	.23
Middle (M40‖) and High (T20¶)	28.0	0.43 (0.19–0.98)		39.4	0.65 (0.33–1.30)	
Familial support						
Good	36.1	0.34 (0.08–1.50)	.14	42.2	0.49 (0.16–1.46)	.19
Poor	62.5	2.95 (0.67–13.10)		60.0	2.05 (0.69–6.14)	
B. Working condition						
Working away from home						
Yes	29.5	0.53 (0.23–1.20)	.13	57.6	2.05 (0.92–4.57)	.08
No	44.3	1.89 (0.83–4.31)		39.8	0.49 (0.22–1.08)	
Workplace support						
Good	30.8	0.31 (0.12–0.76)	**.009**	43.0	0.80 (0.36–1.81)	.60
Poor	59.3	3.27 (1.32–8.10)		48.4	1.24 (0.55–2.79)	
C. Covid-19 related events						
Quarantined as close contacts						
Yes	39.7	1.36 (0.53–2.99)	.60	48.8	1.68 (0.81–3.50)	.16
No	34.4	0.79 (0.33–1.89)		36.2	0.59 (0.29–1.24)	

Factors from sociodemographic background, working condition and Coronavirus disease 2019 (Covid-19) related events were analyzed separately among healthcare workers (HCWs) and non-healthcare workers (non-HCWs).

*P* value < .05: statistical significance.

* Healthcare workers.

† Non-healthcare workers.

‡ Including single, divorced and widowed individuals.

§ Monthly income < RM 5740 or 1214.2 USD.

‖ Monthly income ≤ RM 11919 or 2521.2 USD.

¶ Monthly income ≥ RM 11920 or 2521.4 USD.

Younger age (45.6% vs 21.6%, *P* = .02, OR 3.04, 95% CI 1.21–7.60) and poor workplace support (59.3% vs 29.5%, *P* = .01, OR 3.48, 95% CI 1.40–8.63) was significantly associated with depression symptoms among HCWs (Table 4). In terms of marital status, over half of the respondents were married. 54.5% of single HCWs and 47.7% of single non-HCWs were having depression symptoms, which was significantly higher than married individuals in both HCWs (*P* = .01, OR 2.91, 95% CI 1.24–6.84) and non-HCWs (*P* = .03, OR 2.26, 95% CI 1.07–4.80). Similarly, non-HCWs with higher educational attainment were more likely to have depression symptoms (44.1% vs 13.2%, *P* < .001, OR 2.51, 95% CI 1.12–5.64). Other associated factors identified for non-HCWs were poor family support (*P* = .03, OR 3.20, 95% CI 1.06–9.66), working away from home (*P* = .02, OR 2.53, 95% CI 1.13–5.68) and history of being quarantined as Covid-19 close contact (*P* = .04, OR 2.34, 95% CI 1.05–5.22). Meanwhile, factors such as gender were not significantly associated with anxiety and depression symptoms among both groups (Table 4).

**Table 4:** Associated factors for depression symptoms.

	HCWs* (n = 105)			Non-HCWs† (n = 131)		
	Prevalence of possible depression (%)	OR (95% CI)	*P* value	Prevalence of possible depression (%)	OR (95% CI)	*P* value
A. Sociodemographic						
Gender						
Male	34.5	0.85 (0.35–2.09)	.73	30.4	0.72 (0.34–1.56)	.41
Female	38.2	1.17 (0.48–2.87)		37.6	1.38 (0.64–2.97)	
Age						
≤40	45.6	3.04 (1.21–7.60)	**.02**	37.5	1.20 (0.58–2.47)	.71
>40	21.6	0.40 (0.13–0.82)		33.3	0.83 (0.40–1.72)	
Marital status						
Single‡	54.5	2.91 (1.24–6.84)	**.01**	47.7	2.26 (1.07–4.80)	**.03**
Married	29.2	0.34 (0.15–0.81)		28.7	0.44 (0.21–0.94)	
Education						
Lower level	16.7	0.30 (0.06–1.46)	.12	13.2	0.19 (0.07–0.54)	**<.001**
Higher level	39.8	3.30 (0.68–15.94)		44.1	2.51 (1.12–5.64)	
Income						
Low (B40§)	43.6	1.81 (0.81–4.05)	.15	38.3	1.30 (0.63–2.66)	.48
Middle (M40‖) and High (T20¶)	30	0.55 (0.25–1.24)		32.4	0.77 (0.38–1.58)	
Familial support						
Good	35.1	0.32 (0.07–1.44)	.12	31.9	0.31 (0.10–0.94)	**.03**
Poor	62.5	3.09 (0.70–13.72)		60.0	3.20 (1.06–9.66)	
B. Working condition						
Working away from home						
Yes	36.4	0.94 (0.42–2.11)	.89	51.5	2.53 (1.13–5.68)	**.02**
No	37.7	1.06 (0.47–2.37)		29.6	0.40 (0.18–0.89)	
Workplace support						
Good	29.5	0.29 (0.12–0.71)	**.006**	35.0	0.98 (0.42–2.27)	.96
Poor	59.3	3.48 (1.40–8.63)		35.5	1.02 (0.44–2.37)	
C. Covid-19 related events						
Quarantined as close contact						
Yes	39.7	1.45 (0.60–3.50)	.41	41.7	2.34 (1.05–5.22)	**.04**
No	31.3	0.69 (0.29–1.67)		23.4	0.43 (0.19–0.95)	

Factors from sociodemographic background, working condition and Coronavirus disease 2019 (Covid-19) related events were analyzed separately among healthcare workers (HCWs) and non-healthcare workers (non-HCWs).

*P* value < .05: statistical significance.

* Healthcare workers.

† Non–healthcare workers.

‡ Including single, divorced and widowed individuals.

§ Monthly income < RM 5740 or 1214.2 USD.

‖ Monthly income ≤ RM 11919 or 2521.2 USD.

¶ Monthly income ≥ RM 11920 or 2521.4 USD.

## 4. Discussion

Covid-19 pandemic posted a great mental burden across the globe, affecting the society in various ways. A local study conducted during the third wave of the pandemic reported 25.1% prevalence of severe depression, which was higher compared to our study (2.9% among HCWs and 0.8% among non-HCWs).^[7]^ However, their 34.1% prevalence of mild to moderate anxiety was similar to our study.^[7]^ Specific to HCWs, the prevalence of depression symptoms among our participants (37.3%) was similar to a Kelantan study where 27.5% of frontline HCWs and 37.7% of non-frontline HCWs exhibited depressive symptoms (HADS > 8).^[32]^ However, our prevalence was notably lower than that of a Turkey study, where 51.6% of HCWs experienced anxiety symptoms and 64.7% experienced depressive symptoms.^[13]^ Our overall findings were comparable to a study in China, which reported GAD symptoms in 35.1% and depressive symptoms in 20.1% of participants.^[9]^ This was also consistent with a systematic review where 23.2% and 22.8% prevalence of anxiety and depression were retrieved from meta-analysis.^[14]^

Our study did not report significant difference between HCWs and non-HCWs in terms of the prevalence of depression and anxiety symptoms. This is supported by a 2021 Malaysia study and a 2022 Pakistan study, in which both reported similar prevalence of mental health issues among these 2 study groups.^[32,36]^ This may mean that the degree of mental burden brought by Covid-19 to both HCWs and non-HCWs is comparable. Despite not dealing with Covid-19 cases directly, non-HCWs can be predisposed to mental health issues due to various socioeconomics factors. A recent longitudinal study across 3 phases of Covid-19 reported reducing trend of emotional exhaustion as Covid-19 progresses despite having similar rates of mental health burden during phase 1.^[41]^ This suggested that the outcome is related to the time of data collection. Our study period was at the end of phase 1, hence the similar prevalence between HCWs and non-HCWs can only represent that phase of Covid-19. As the pandemic progresses, the discrepancy might be extrapolated alongside with the increased burnout rates among HCWs, which warrants follow-up studies.

Younger age and female gender have been well known factors associated with mental health conditions among HCWs^[13]^ and general population.^[9,10,42,43]^ A systematic review in 2020 found that females and younger age groups have more major depressive disorder and anxiety disorder than their counterparts.^[44]^ It may be due to more frequent use of social media among the younger population, leading to worries and distress triggered by inaccurate information online. Since we have limited respondents from the older age group, the generalizability of our data remains uncertain. Future studies involving a more balanced distribution of younger and older population will be helpful to evaluate this factor further.

Being single is associated with depression symptoms among both HCWs and non-HCWs. As supported by a Turkey study, single HCWs reported higher scores of Depression Anxiety and Stress Scale-21 (DASS-21).^[13]^ This is the same for their general population where single individuals have significantly higher prevalence of depression than married individuals (62.9% vs 37.1%, *P* = .001).^[43]^ Our study categorized individuals who are divorced and widowed as being single as well. This group of individuals lack support and companionship, which may worsen their mental health. In contrast, married HCWs in China are more likely to have depression.^[45]^ The contradiction might be contributed by the different life stressors faced by single and married individuals respectively. More studies are needed to evaluate the context in depth.

In addition, poor workplace support is significantly associated with anxiety and depression symptoms among HCWs. This is supported by an international cross-sectional study that established the influence of workplace support on the mental health status of front-line HCWs.^[34]^ Poorly supported HCWs are facing more psychological violence, workplace bullying, poor human relations and interpersonal conflict.^[35]^ All these have contributed to the development of anxiety and depression among HCWs.^[35]^

Our study reported higher prevalence of mental health symptoms among non-HCWs with higher educational attainment. Whilst it is true that students with higher educational levels sustained more stress during Covid-19 era, it might not be the case for those in the working field.^[46]^ Majority of recent studies reported higher prevalence of mental health issues in populations with lower educational attainment.^[47,48]^ These individuals are more likely to have financial insecurity, thus precipitating more psychological distress.^[49]^ In contrast, highly educated individuals have a better insight and awareness towards mental health.^[49]^ Being more health-conscious may lead to certain individuals constantly worrying about the latest progress of the pandemic, provoking more negative thoughts and emotions. This inconsistent finding from our study might be attributed to the rationale above. Nevertheless, most non-HCWs in our study are highly educated, thus causing a certain degree of sampling bias. Further studies are required to investigate the mental health impact in respective educational levels.

Several other association factors are found for either HCWs or non-HCWs. For instance, HCWs with low income (B40, monthly income less than 1214.2 USD) are at greater risk of experiencing anxiety symptoms. Financial discrepancies exist even within the healthcare system, with HCWs from different job categories earning different monthly incomes. HCWs have to deal with extra workload and longer working hours during the pandemic. The lack of increment in income has likely increased their levels of stress and depression.^[50]^ This is in line with a study done in Ethiopia where HCWs who earned 4000 Ethiopian birrs per month were 12.56 times more likely to report anxiety symptoms compared to those with a monthly salary of 8000 Ethiopian birrs or more.^[51]^

In our study, working away from home is a significant factor associated with depression symptoms among non-HCWs. An article published by Prithwiraj and Ohchan^[52]^ explained that individuals working away from home have poorer workplace performance in the long term. They are unable to visit family and friends, and the social isolation is postulated to be negatively affecting their mental health status as well.^[52]^ This is relatable to another factor established in our study, where non-HCWs with poor familial support are more at risk of depression. Family is the most crucial type of social support for people.^[53]^ They play vital roles in improving self-esteem and providing emotional support to those suffering from severe psychological distress.^[53]^ In contrast, no significant association was identified between the level of familial support and prevalence of anxiety or depression symptoms among HCWs. This could be due to higher levels of resilience among HCWs who have been working in high-risk environments, dealing with challenging situations even before the emergence of Covid-19.^[54]^ This is supported by an Iran study which found that resilient HCWs were less likely to suffer from mental health conditions.^[55]^

Lastly, history of being quarantined as Covid-19 close contact is also an associating factor for depression symptoms among non-HCWs. This is consistent with 3 meta-analyses which discovered a positive correlation between quarantine and depression.^[56,57]^ It was postulated that quarantine changes a person’s diet and sleep which could worsen the severity of depression.^[56]^ Fear of infection and financial concerns also played a role in contributing to poor mental health status.^[58,59]^

These findings highlight the importance of early mental health promotion for the general public. Reasonable workplace adjustments and better welfare could be provided to HCWs since adequate workplace support is a key factor for good mental health. This can include informational, instrumental, organizational, emotional and psychological support which have each played vital role in addressing mental health issues among HCWs during infectious disease outbreaks.^[60]^ For non-HCWs, more comprehensive and easily accessible mental health support services should be provided at their workplace, giving the same emphasis as their physical wellbeing. Dissemination of false or misleading health information should be strictly controlled by the authority to avoid unnecessary worries.

## 5. Study limitations

This study has some limitations. Despite GAD 7 and PHQ 9 being highly sensitive and specific, proper assessment by mental health physicians is vital to diagnose mental health disorders among the individuals.^[37,38]^ Hence, participants were classified as having “anxiety symptoms,” “possible anxiety,” “depression symptoms” or “possible depression” rather than being diagnosed with anxiety or depression in our study. The cross-sectional study nature could not deduce a clear causal relationship between the variables and the prevalence of mental health disorders. Majority of participants are highly educated, thus further studies across population with different literacies are needed. The sample size is smaller than estimated, which could affect the generalisability of the results to the Johor population. Further large-scale studies with a careful consideration of confounding factors are warranted.

## 6. Conclusion

Around one third of the participants are having anxiety or depression symptoms in our study. There is no significant difference in the prevalence of anxiety and depression symptoms among HCWs and non-HCWs. Specific to HCWs, the factors significantly associated with anxiety symptoms are poor workplace support and low income, while those identified for depression symptoms included younger age, single status, and poor workplace support. As for non-HCWs, higher educational level is associated with anxiety and depression symptoms. Being single, working away from home, poor familial support, and history of being quarantined as Covid-19 close contact are the factors associated with depression symptoms among non-HCWs.

This study suggests that both HCWs and non-HCWs are still having a considerable level of psychological distress even with the reducing trend of Covid-19 burden nowadays. The mental health status of both groups should be taken care of, with attention paid to the specific associated factors identified among HCWs and non-HCWs. Policies to improve workplace support and welfare especially among HCWs might be beneficial. More studies are needed to evaluate the association in depth.

## Acknowledgments

We sincerely thank Dr Soe Moe, Professor and Head of Department, Community Medicine, Manipal University College Malaysia, for her expert advice and guidance in statistical analysis. We would also like to thank all the HCWs and non-HCWs for their participation in our study.

## Author contributions

**Conceptualization:** Jing Wen Wong, Jun Hui Tan, Ruth Elizabeth Abraham, Shareen Nisha Jauhar Ali, Si Yin Kok, Han Ni.

**Data curation:** Jing Wen Wong, Jun Hui Tan, Ruth Elizabeth Abraham, Shareen Nisha Jauhar Ali, Si Yin Kok.

**Formal analysis:** Jing Wen Wong, Jun Hui Tan, Ruth Elizabeth Abraham, Shareen Nisha Jauhar Ali, Si Yin Kok.

**Investigation:** Henry Chor Lip Tan, Jih Huei Tan.

**Methodology:** Jing Wen Wong, Jun Hui Tan.

**Project administration:** Jing Wen Wong, Jun Hui Tan, Ruth Elizabeth Abraham, Shareen Nisha Jauhar Ali, Si Yin Kok.

**Resources:** Jih Huei Tan, Han Ni.

**Supervision:** Henry Chor Lip Tan, Jih Huei Tan, Han Ni.

**Validation:** Henry Chor Lip Tan, Jih Huei Tan, Han Ni.

**Visualization:** Jing Wen Wong, Han Ni.

**Writing – original draft:** Jing Wen Wong, Jun Hui Tan, Ruth Elizabeth Abraham, Shareen Nisha Jauhar Ali, Si Yin Kok.

**Writing – review & editing:** Jing Wen Wong.
